# Anxiety Disorders in the Perinatal Period

**DOI:** 10.1192/j.eurpsy.2022.97

**Published:** 2022-09-01

**Authors:** L. Mcdonald

**Affiliations:** Royal College of Psychiatrists, Dept Of Professional Standards, London, United Kingdom

**Keywords:** Perinatal, maternal OCD, Anxiety

## Abstract

Anxiety disorders are common in pregnancy and in the post-natal period. This presentation will focus on how anxiety disorders may present in the perinatal period, the need for accurate and timely diagnosis, and barriers to diagnosis and barriers for women in accessing appropriate care and treatment.

**Disclosure:**

No significant relationships.

